# VO-Notches Subjected to Tension–Torsion Loading: Experimental and Theoretical Fracture Study on Polymeric Samples

**DOI:** 10.3390/polym15112454

**Published:** 2023-05-25

**Authors:** Hossein Talebi, Mohsen Askari, Majid Reza Ayatollahi, Sergio Cicero

**Affiliations:** 1 Fatigue and Fracture Research Laboratory, Center of Excellence in Experimental Solid Mechanics and Dynamics, School of Mechanical Engineering, Iran University of Science and Technology, Tehran 16846, Iran; hsn.talebi75@gmail.com (H.T.); askari.mosn@gmail.com (M.A.); 2 LADICIM, Departamento de Ciencia e Ingeniería del Terreno y de los Materiales, Universidad de Cantabria, 39005 Santander, Spain

**Keywords:** fracture behavior, V-shaped notch with end hole, maximum tangential stress (MTS), mean stress (MS), mixed-mode I/III loading

## Abstract

In this research, the fracture behavior of brittle specimens weakened by V-shaped notches with end holes (VO-notches) is studied. First, an experimental investigation is conducted to evaluate the effect of VO-notches on fracture behavior. To this end, VO-notched samples of PMMA are made and exposed to pure opening mode loading, pure tearing mode loading, and some combinations of these two loading types. As part of this study, samples with end-hole radii of 1, 2, and 4 mm are prepared to determine the effect of the notch end-hole size on the fracture resistance. Second, two well-known stress-based criteria, namely the maximum tangential stress (MTS) criterion and the mean stress (MS) criterion, are developed for VO-shaped notches subjected to mixed-mode I/III loading, also determining the associated fracture limit curves. A comparison between the theoretical and the experimental critical conditions indicates that the resulting VO-MTS and VO-MS criteria predict the fracture resistance of VO-notched samples with about 92% and 90% accuracy, respectively, confirming their capacity to estimate fracture conditions.

## 1. Introduction

With the exception of some very limited cases in which fracture occurrence is desirable, such as the breakage of massive rocks in mining-related activities, it is generally a priority to prevent the initiation and growth of cracks in engineering components and structures. The main reason is that cracks lead to stress concentrations and, eventually, may cause the structure to fail at substantially lower loads. In spite of this, the use of notches of different shapes is usually inevitable since they are required for assembling components and parts, accessing special components in the structure, transferring power from one component to another, etc. Like cracks, notches also create stress concentrations in the structures and are always prone to cracking, making it essential to assess the strength of notched structures in order to ensure their reliability and integrity. By estimating the failure load of a notched structure accurately, engineers can design such a structure to ensure that it can withstand the applied loads without cracking or fracture.

Depending on the particular application, notches with different shapes may be employed in the structure being analyzed, such as U-, V-, or O-shaped notches. When a short crack initiates from the notch edge, it is susceptible to growth; therefore, some methods have been suggested to prevent the additional growth of the initiated crack. For example, dealing with a V-shaped notch, it is recommended to drill a hole with a radius equal to the crack length at the tip of the notch. As a result, the original V-shaped notch becomes a V-shaped notch with an end hole, usually referred to as a VO-notch. Manifestly, the stress gradient in the repaired notch, i.e., the VO-notch, is lower than that in the original notch, as the shape of the tip of the original notch is changed into an O. However, it is not always clear without stress analysis whether the original V-notch or the resulting VO-notch generates a higher stress concentration, as the latter has a greater size, but the former has a sharper tip. Hence, the fracture analysis of the resulting VO-notch is essential to ensure the safety and integrity of a repaired notched structure.

Fracture in brittle materials, such as glass, ceramics, graphite, and some polymers, occurs abruptly without or with negligible plastic deformations. The presence of a stress concentrator, such as a notch, in these brittle materials makes them susceptible to crack initiation and, eventually, to sudden fracture. Therefore, it is essential to accurately estimate the notch fracture toughness of brittle materials by means of appropriate fracture models. These models must be carefully verified by applying them to experimental results achieved from the fracture testing of notched specimens.

There are a variety of brittle/quasi-brittle polymers, among which polymethyl-methacrylate (PMMA) is one of the most well-known. Fracture assessment of PMMA, especially when notches are introduced in it, is very important because of its widespread engineering applications. In addition, the fact that this material is available in various shapes, such as sheets of different thicknesses, together with its capacity to be machined, also makes PMMA ideal for brittle fracture testing. A number of papers can be found in the literature related to the fracture assessment of PMMA in the presence or absence of stress concentrators (e.g., [1,2,3,4,5,6,7]).

Cracked and notched components may experience various loading conditions, such as pure mode I (opening mode) loading, pure mode II (sliding mode) loading, and pure mode III (tearing mode) loading, or a combination of these modes, generally known as mixed-mode loading. Regarding brittle fracture estimation under mode I loading, which is the simplest loading mode, the strain energy density (SED) [8,9], maximum tangential stress (MTS) [10], cohesive zone model (CZM) [11], generalized J-integral [12,13], and finite fracture mechanics (FFM) [14,15] criteria are available, among others. These criteria have also been extended to mixed-mode I/II fracture problems, as is the case of [16,17,18] for the SED criterion, [19,20] for the point stress (PS) and mean stress (MS) approaches, and [21] for the FFM criterion.

The criteria mentioned above are relevant to in-plane loading conditions. If mode III loading is applied solely or in combination with mode I loading, the loading condition is pure mode III loading or mixed-mode I/III loading. In comparison to in-plane loading, fewer fracture studies have been conducted on out-of-plane loading. Zheng et al. [22] proposed a criterion based on the combination of the use of normal stress and Griffith energy to analyze pure mode III loading. Berto et al. [23,24] applied the SED criterion to U-shaped and V-shaped notches under pure mode III loading. Saboori et al. [25] rotated the in-plane stress distribution around a round-tip V-shaped notch with the help of the rotation matrix and generalized mixed-mode I/II loading to mixed-mode I/II/III loading. Then, they used the PS and MS criteria to predict the fracture of notched components subjected to tension–torsion loading. Ayatollahi and Saboori [26] extended the generalized MTS criterion to mixed-mode I/II/III loading. They found that the T-stress term is more effective in mode II loading compared to mode III loading. Chang et al. [27] also proposed a criterion based on the concept of maximum potential energy release rate (MPERR) for the general mixed-mode I/II/III loading.

In order to provide mixed-mode I/III loading conditions in fracture experiments, some test specimens with different geometries have already been proposed. For instance, Ayatollahi and Saboori [28] presented a new fixture that could produce mixed-mode I/III loading conditions with various combinations of mode I and mode III loadings at the crack/notch neighborhood simply by applying a remote tensile load. The edge-notched diametrically compressed disc (ENDC) and the edge-notched disc bend (ENDB) specimens are two samples suggested for fracture testing under mixed-mode I/III loading. Although they both have similar geometries, the loading and boundary conditions of the two samples are different [29,30,31]. In another study, cylindrical notched specimens were exposed to combined tension–torsion loading in order to provide mixed-mode I/III loading conditions [32]. Then, by introducing spiral notches with different pitch angles and applying pure torsion, Wang [33] created mixed-mode I/III loading conditions on the cylindrical specimens.

In this study, the brittle fracture of VO-notched specimens made of PMMA was assessed under pure mode I, mixed-mode I/III, and pure mode III loadings. According to the experimental observations, the PMMA examined showed brittle behavior with an almost linear elastic stress–strain curve at room temperature. Thus, in Section 2, the MTS and MS criteria, which are two well-known fracture models of linear elastic notch fracture mechanics (LENFM), are theoretically formulated, establishing the tools to define the corresponding fracture limit curves. Section 2 also presents the experimental campaign of this research (Section 2.4), in which fracture tests were conducted on un-notched, pre-cracked, and VO-notched specimens, and the finite element (FE) analyses (Section 2.5), which were necessary to numerically compute the fracture parameters of the VO-notches, are described in detail. Section 3 gathers the different results and provides a comparison between the notch fracture toughness (NFT) predictions of the two fracture models and the experimental data, with a discussion about the results achieved included in Section 4. Lastly, some concluding remarks are presented in Section 5.

## 2. Materials and Methods

### 2.1. Definition of Stress Fields in VO-Notches

**Figure 1:**
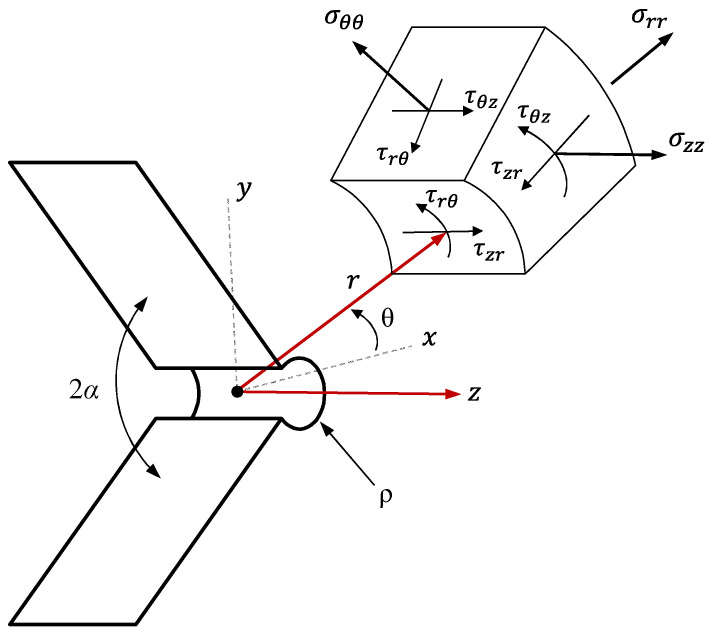
VO-notch stress components in the cylindrical coordinate system.

In order to employ fracture criteria to derive theoretical predictions of the fracture behavior, it is essential to know the stress distribution surrounding the notch. The stress distribution around VO-shaped notches for different opening angles was defined by Zappalorto and Lazzarin in [34]. As mentioned above, the purpose of the present study was to investigate the fracture of VO-notched brittle components exposed to mixed-mode I/III loading conditions. The equations related to the stress field of VO-notches for pure mode I and pure mode III loadings are as follows:

(1) $$\begin{array}{c} \sigma_{\theta \theta }=\frac{K_{I}^{\mathrm{VO},\rho }}{\sqrt{2\pi }}\frac{r^{\lambda_{1}-1}}{\left(1+\lambda_{1}\right)+\varphi_{1}\left(\gamma \right)}\{\cos\left(1-\lambda_{1}\right)\theta \left[\left(1+\lambda_{1}\right)+\psi_{11}\left(\theta \right){\left(\frac{\rho }{r}\right)}^{2\lambda_{1}}+\psi_{12}\left(\theta \right)\chi_{11}\left(\theta \right){\left(\frac{\rho }{r}\right)}^{2\lambda_{1}+1}\right]\\ +\varphi_{1}\left(\gamma \right)\cos\left(1+\lambda_{1}\right)\theta \left[1+\left(1-\lambda_{1}\right){\left(\frac{\rho }{r}\right)}^{2\lambda_{1}}+\left(2+\lambda_{1}\right){\left(\frac{\rho }{r}\right)}^{2\left(\lambda_{1}+1\right)}\right]\} \end{array}$$

(2) $$\begin{array}{c} \sigma_{\mathrm{rr}}=\frac{K_{I}^{\mathrm{VO},\rho }}{\sqrt{2\pi }}\frac{r^{\lambda_{1}-1}}{\left(1+\lambda_{1}\right)+\varphi_{1}\left(\gamma \right)}\{\cos\left(1-\lambda_{1}\right)\theta \left[\left(3-\lambda_{1}\right)-\psi_{11}\left(\theta \right){\left(\frac{\rho }{r}\right)}^{2\lambda_{1}}-\psi_{12}\left(\theta \right)\chi_{11}\left(\theta \right){\left(\frac{\rho }{r}\right)}^{2\lambda_{1}+1}\right]\\ +\varphi_{1}\left(\gamma \right)\cos\left(1+\lambda_{1}\right)\theta \left[\left(3+\lambda_{1}\right){\left(\frac{\rho }{r}\right)}^{2\lambda_{1}}-1-\left(2+\lambda_{1}\right){\left(\frac{\rho }{r}\right)}^{2\left(\lambda_{1}+1\right)}\right]\} \end{array}$$

(3) $$\sigma_{\mathrm{zz}}=\begin{array}{ll} 0 & \mathrm{Plane}\;\mathrm{stress}\\ \upsilon \left(\sigma_{\theta \theta }+\sigma_{\mathrm{rr}}\right) & \mathrm{Plane}\;\mathrm{Strain} \end{array}$$

(4) $$\tau_{\theta z}=\frac{K_{\mathrm{III}}^{\mathrm{VO},\rho }}{\sqrt{2\pi }}r^{\lambda_{3}-1}\cos\left(\frac{2\theta }{3}\right)\left[1+{\left(\frac{\rho }{r}\right)}^{2\lambda_{3}}\right]$$

(5) $$\tau_{\mathrm{zr}}=\frac{K_{\mathrm{III}}^{\mathrm{VO},\rho }}{\sqrt{2\pi }}r^{\lambda_{3}-1}\sin\left(\frac{2\theta }{3}\right)\left[1-{\left(\frac{\rho }{r}\right)}^{2\lambda_{3}}\right]$$

where K_I_^VO,ρ^ and K_III_^VO,ρ^ stand for the mode I and mode III VO-notch stress intensity factors (VO-NSIFs), respectively. The NSIF is an indicator of the stress concentration in the vicinity of the notch. It is used to compute the stress level in a localized area of a notched component and is a crucial factor when determining the fracture strength and the fatigue life of an engineering structure that contains a notch. As illustrated in Figure 1, σ_θθ_, σ_rr_, and τ_rθ_ are the tangential, radial, and in-plane shear stresses, respectively. τ_zθ_ and τ_zr_ are the out-of-plane shear stresses, and σ_zz_ denotes normal stress along the Z direction. ρ indicates the VO-notch end-hole radius and in Equation (3), υ stands for the Poisson’s ratio. ψ_11_(θ) and ψ_12_ (θ) χ_11_(θ) are also two auxiliary parameters, which are defined as follows:

(6) $$\psi_{11}\left(\theta \right)=\frac{\left[2\sin\lambda_{1}\theta \cos\left(\lambda_{1}-1\right)\theta +\left(1-\lambda_{1}\right)\sin\left(2\lambda_{1}-1\right)\theta \right]}{\sin\theta }$$

(7) $$\psi_{12}\left(\theta \right)\chi_{11}\left(\theta \right)=\frac{2\left(2-\lambda_{1}\right)}{1+\frac{\tan\lambda_{1}\theta }{\tan\left(1-\lambda_{1}\right)\theta }}$$

Due to the absence of mode II loading, only the out-of-plane shear deformations are combined with the tensile deformations and, thus, the in-plane fracture angle θ can be considered zero. The values of the auxiliary parameters for θ = 0, together with those of the dimensionless parameters λ_1_, λ_3_, and φ_1_ for the VO-notch with an opening angle of 2α = 90°, are given in Table 1. The values of the dimensionless and auxiliary parameters for VO-notched specimens with different notch opening angles and subjected to in-plane and out-of-plane loadings are given in reference [34].

Using the values presented in Table 1, the equations of the stress field can be simplified into

(8) $$\sigma_{\theta \theta }=\frac{K_{I}^{\mathrm{VO},\rho }}{\sqrt{2\pi }}\frac{r^{\lambda_{1}-1}}{2.38}\left[2.38+{\left(\frac{\rho }{r}\right)}^{2\lambda_{1}}\left(1.5+1.33\left(\frac{\rho }{r}\right)+2.13{\left(\frac{\rho }{r}\right)}^{2}\right)\right]$$

(9) $$\sigma_{\mathrm{rr}}=\frac{K_{I}^{\mathrm{VO},\rho }}{\sqrt{2\pi }}\frac{r^{\lambda_{1}-1}}{2.38}\left[1.61+{\left(\frac{\rho }{r}\right)}^{2\lambda_{1}}\left(1.84-1.33\left(\frac{\rho }{r}\right)-2.13{\left(\frac{\rho }{r}\right)}^{2}\right)\right]$$

(10) $$\tau_{\theta z}=\frac{K_{\mathrm{III}}^{\mathrm{VO},\rho }}{\sqrt{2\pi }}r^{\lambda_{3}-1}\left[1+{\left(\frac{\rho }{r}\right)}^{2\lambda_{3}}\right]$$

In order to calculate the mode I and mode III VO-NSIFs for a given load, Equations (11) and (12) can be employed:

(11) $$K_{I}^{\mathrm{VO},\rho }=\frac{2.38\sqrt{2\pi }\sigma_{\theta \theta }}{r^{\lambda_{1}-1}\left[2.38+{\left(\frac{\rho }{r}\right)}^{2\lambda_{1}}\left(1.5+1.33\left(\frac{\rho }{r}\right)+2.13{\left(\frac{\rho }{r}\right)}^{2}\right)\right]}$$

(12) $$K_{\mathrm{III}}^{\mathrm{VO},\rho }=\frac{\sqrt{2\pi }\tau_{\theta z}}{r^{\lambda_{3}-1}\left[1+{\left(\frac{\rho }{r}\right)}^{2\lambda_{3}}\right]}$$

The stress components of the element in a cylindrical coordinate system can be written as a stress tensor S.

(13) $$S=\left[\begin{array}{ccc} \sigma_{\mathrm{rr}} & \tau_{r\theta } & \tau_{\mathrm{rz}}\\ \tau_{r\theta } & \sigma_{\theta \theta } & \tau_{\theta z}\\ \tau_{\mathrm{rz}} & \tau_{\theta z} & \sigma_{\mathrm{zz}} \end{array}\right]$$

Due to the presence of tearing mode loading, out-of-plane shear deformations contribute to fracture. Thus, in order to include the participation of both mode I and mode III loadings together in the stress components, the rotation matrix R_r_(ϕ) is used as presented in Equation (14) [25].

(14) $$R_{r}\left(\phi \right)=\left[\begin{array}{ccc} 1 & 0 & 0\\ 0 & \cos\phi & \sin\phi \\ 0 & -\sin\phi & \cos\phi \end{array}\right]$$

**Figure 2:**
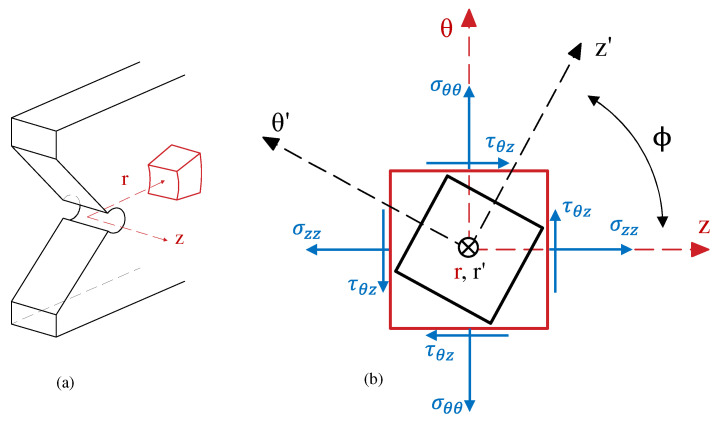
Views of the element in the cylindrical system of reference. (**a**) Three-dimensional and (**b**) θ-Z plane.

The R_r_ matrix rotates the stress elements around the r axis by ϕ (see Figure 2). Equation (15) provides a formula for calculating the new stress components in the rotated cylindrical system of reference (r′, θ′, z′).

(15) $$S^{\prime }=R_{r}{S\;R}_{r}{}^{T}$$

Introducing Equations (13) and (14) into Equation (15) gives

(16) $$S^{\prime }=\left[\begin{array}{ccc} \sigma_{\mathrm{rr}} & \tau_{r\theta^{\prime }} & \tau_{rz^{\prime }}\\ \tau_{r\theta^{\prime }} & \sigma_{\theta^{\prime }\theta^{\prime }} & \tau_{\theta^{\prime }z^{\prime }}\\ \tau_{rz^{\prime }} & \tau_{\theta^{\prime }z^{\prime }} & \sigma_{z^{\prime }z^{\prime }} \end{array}\right]$$

According to Equation (16), the mixed-mode I/III tangential stress component for VO-notches is obtained as:

(17) $$\begin{array}{ll} \sigma_{\theta^{\prime }\theta^{\prime }}\left(r,\phi \right) & =\cos^{2}\phi \sigma_{\theta \theta }+\sin^{2}\phi \sigma_{\mathrm{zz}}-\sin2\phi \sigma_{\theta z}\\ & =\frac{1}{\sqrt{2\pi }}\left[{X\;r}^{\lambda_{1}-1}K_{I}^{\mathrm{VO},\rho }\cos^{2}\phi +{Z\;r}^{\lambda_{1}-1}K_{I}^{\mathrm{VO},\rho }\upsilon \sin^{2}\phi -{Y\;r}^{\lambda_{3}-1}K_{\mathrm{III}}^{\mathrm{VO},\rho }\sin2\phi \right] \end{array}$$

where

(18) $$X=1+{\left(\frac{\rho }{r}\right)}^{2\lambda_{1}}\left(0.63+0.56\left(\frac{\rho }{r}\right)+0.89{\left(\frac{\rho }{r}\right)}^{2}\right)$$

(19) $$Y=1+{\left(\frac{\rho }{r}\right)}^{2\lambda_{3}}$$

(20) $$Z=1.68+1.41{\left(\frac{\rho }{r}\right)}^{2\lambda_{1}}$$

Since the theoretical failure criteria employed in this study are stress-based and the only stress component needed for fracture prediction is the tangential stress (σ_θθ_), the other stress components are not discussed hereinafter.

### 2.2. MTS Criterion

The MTS criterion states that a crack nucleates from a point on the notch boundary where the tangential stress is the maximum. Normally, the maximum load that a given notched component can withstand is achieved at the onset of crack nucleation from the notch edge. In notched members made of brittle materials, the final fracture occurs abruptly after crack nucleation, and thus, the notch fracture toughness may be defined as the capability of a notch to withstand crack initiation. The MTS criterion also states that brittle fracture happens as soon as the value of tangential stress at the critical distance (r_c,VO_ for VO-notches) in front of the notch edge reaches the material critical stress (σ_c_). Additionally, σ_c_ is usually considered a material property, and, in brittle materials, its value is assumed to be equal to the ultimate tensile strength σ_u_.

**Figure 3:**
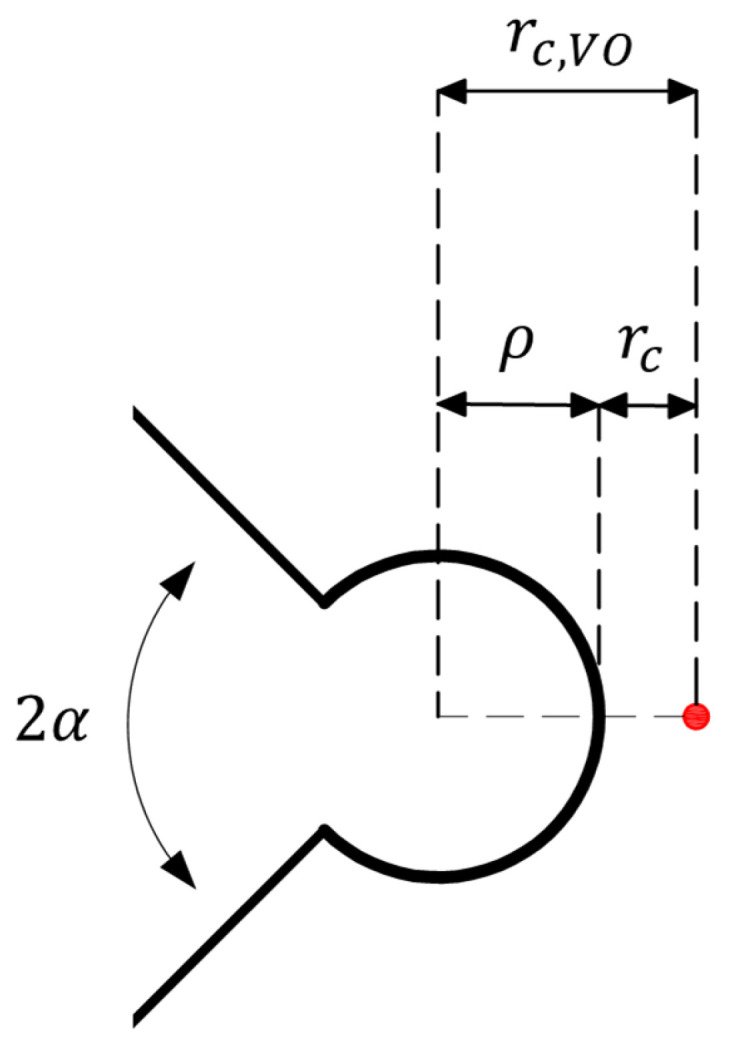
Critical distances of the VO-notch MTS (VO-MTS) criterion.

As shown in Figure 3, r_c,VO_ is measured from the center of the notch hole, and calculated using Equation (21).

(21) $$r_{c,\mathrm{VO}}=r_{c}+\rho$$

r_c_ is the critical distance that is measured from the edge of the notch and can be obtained for the out-of-plain loading conditions by the following Equation (22) [25]:

(22) $$r_{c}=\frac{1}{2\pi }{\left(\frac{K_{\mathrm{IIIc}}}{\sigma_{u}}\right)}^{2}$$

where K_IIIc_ denotes the mode III fracture toughness of the material.

According to the first assumption of the VO-MTS criterion, the condition of the maximum tangential stress in fracture conditions can be mathematically expressed as:

(23) $$\frac{\partial \sigma_{\theta^{\prime }\theta^{\prime }}}{\partial \phi }{|}_{\phi =\phi_{f},r=r_{c,\mathrm{VO}}}=0$$

(24) $$\frac{\partial^{2}\sigma_{\theta^{\prime }\theta^{\prime }}}{\partial \phi^{2}}{|}_{\phi =\phi_{f},r=r_{c,\mathrm{VO}}}<0$$

Introducing Equation (17) into Equation (23), and dividing both sides of the equation by the VO-notch mode I fracture toughness (K_Ic_^VO,ρ^) gives:

(25) $$\frac{K_{I}^{\mathrm{VO},\rho }}{K_{\mathrm{Ic}}^{\mathrm{VO},\rho }}\left(\upsilon Z^{\prime }-X^{\prime }\right)\sin2\phi_{f}-2\frac{K_{\mathrm{III}}^{\mathrm{VO},\rho }}{K_{\mathrm{Ic}}^{\mathrm{VO},\rho }}r_{C,\mathrm{VO}}^{\lambda_{3}-\lambda_{1}}Y^{\prime }\cos2\phi_{f}=0$$

where

(26) $$X^{\prime }=1+{\left(\frac{\rho }{r_{c.VO}}\right)}^{2\lambda_{1}}(0.63+0.56\left(\frac{\rho }{r_{c.VO}}\right)+0.89{\left(\frac{\rho }{r_{c.VO}}\right)}^{2})$$

(27) $$Y^{\prime }=1+{\left(\frac{\rho }{r_{c.VO}}\right)}^{2\lambda_{3}}$$

(28) $$Z^{\prime }=1.68+1.41\;{\left(\frac{\rho }{r_{c.VO}}\right)}^{2\lambda_{1}}$$

The values of the variables/parameters K_I_^VO,ρ^, K_III_^VO,ρ^, and ϕ_f_ presented in Equation (25) are given in Table 2 under pure opening mode and pure tearing mode.

As reported in Table 2, in pure mode III loading, ϕ_f_ has two values, but only −π⁄4 satisfies Equation (24). Thus, fracture occurs at an angle between 0 and −π⁄4 in mixed-mode I/III loading.

According to the VO-MTS criterion, the second assumption for fracture happening is

(29) $$\sigma_{\theta^{\prime }\theta^{\prime }}\left(r_{c,\mathrm{VO}},\phi_{f}\right)=\sigma_{c}$$

Equations (17) and (29) can be re-written as:

(30) $$\frac{1}{\sqrt{2\pi }}\left[r_{c.\mathrm{VO}}^{\lambda_{1}-1}K_{I}^{\mathrm{VO},\rho }X^{\prime }\cos^{2}\phi_{f}+r_{c,\mathrm{VO}}^{\lambda_{1}-1}K_{I}^{\mathrm{VO},\rho }\upsilon Z^{\prime }\sin^{2}\phi_{f}-r_{c,\mathrm{VO}}^{\lambda_{3}-1}K_{\mathrm{III}}^{\mathrm{VO},\rho }Y^{\prime }\sin2\phi_{f}\right]=\sigma_{c}$$

By applying fracture conditions under pure mode I loading, given in Table 2, to Equation (30):

(31) $$\frac{r_{c,\mathrm{VO}}^{\lambda_{1}-1}}{\sqrt{2\pi }}K_{\mathrm{Ic}}^{\mathrm{VO},\rho }X^{\prime }=\sigma_{c}$$

Introducing the left side of Equation (30) into the right side of Equation (31), and then dividing both sides by $\frac{r_{c,\mathrm{VO}}^{\lambda_{1}-1}}{\sqrt{2\pi }}K_{\mathrm{Ic}}^{\mathrm{VO},\rho }$ gives

(32) $$\frac{K_{I}^{\mathrm{VO},\rho }}{K_{\mathrm{Ic}}^{\mathrm{VO},\rho }}\left(X^{\prime }\cos^{2}\phi_{f}+\upsilon Z^{\prime }\sin^{2}\phi_{f}\right)-r_{c,\mathrm{VO}}^{\lambda_{3}-\lambda_{1}}\frac{K_{\mathrm{III}}^{\mathrm{VO},\rho }}{K_{\mathrm{Ic}}^{\mathrm{VO},\rho }}Y^{\prime }\sin2\phi_{f}=X^{\prime }$$

In both Equations (25) and (32), $K_{I}^{\mathrm{VO},\rho }/K_{\mathrm{Ic}}^{\mathrm{VO},\rho }$ and $r_{c,\mathrm{VO}}^{\lambda_{3}-\lambda_{1}}K_{\mathrm{III}}^{\mathrm{VO},\rho }/K_{\mathrm{Ic}}^{\mathrm{VO},\rho }$ are called the normalized VO-NSIFs. Solving Equations (25) and (32) simultaneously for some arbitrary values of ϕ_f_ between 0 and −π⁄4 provides the corresponding points with coordinates $K_{I}^{\mathrm{VO},\rho }/K_{\mathrm{Ic}}^{\mathrm{VO},\rho }\;$ as the X component and $r_{c,\mathrm{VO}}^{\lambda_{3}-\lambda_{1}}K_{\mathrm{III}}^{\mathrm{VO},\rho }/K_{\mathrm{Ic}}^{\mathrm{VO},\rho }$ as the Y component. Now, connecting such points, the fracture limit curve of the VO-MTS criterion is obtained, which determines the onset of fracture in VO-notched members subjected to mixed-mode I/III loading. Evidently, selecting a larger number of ϕ_f_ values results in a larger number of points on the plane, and thus, a more accurate fracture limit curve is obtained.

### 2.3. MS Criterion

**Figure 4:**
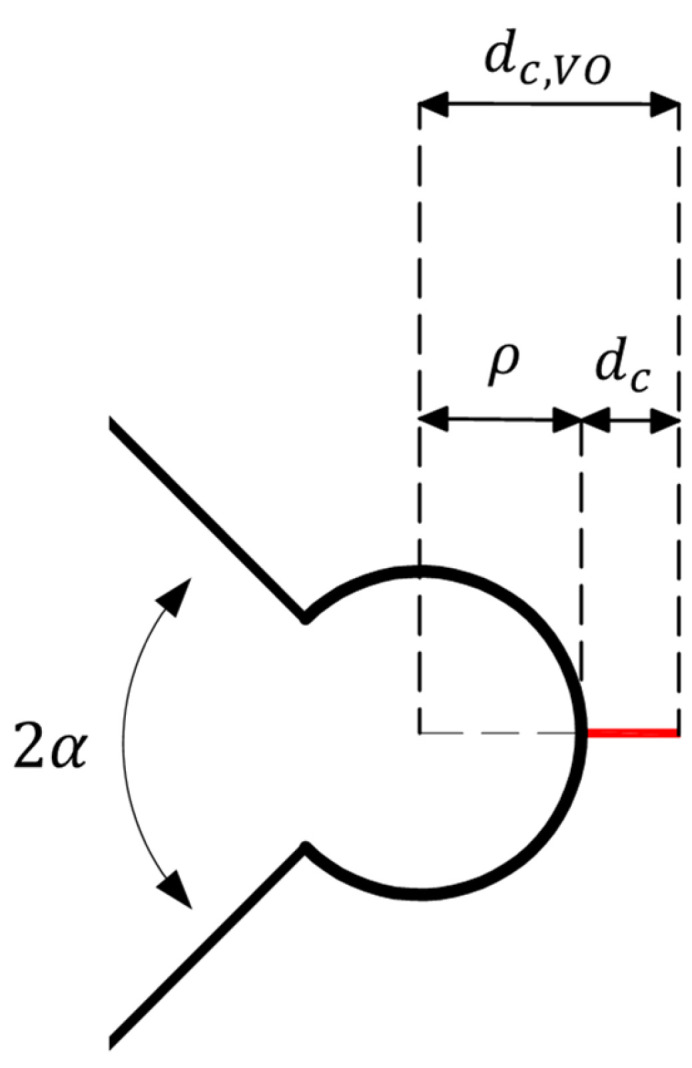
Critical distances of the VO-notch MS (VO-MS) criterion.

In accordance with the MS criterion, brittle fracture will happen if the mean value of the tangential stress over a certain critical distance in front of the edge of notch (e.g., d_c,VO_ for VO-notches) reaches the material critical stress σ_c_. Figure 4 defines d_c,VO_, which can be calculated by Equation (33):

(33) $$d_{c,\mathrm{VO}}=\rho +d_{c}$$

In Equation (33), d_c_ is the critical distance that is measured from the tip of the notch and can be computed for tearing mode loading conditions by Equation (34) [35]:

(34) $$d_{c}=\frac{2}{\pi }{\left(\frac{K_{\mathrm{IIIc}}}{\sigma_{u}}\right)}^{2}$$

As is clear in Equations (22) and (34), the critical distance in both the MTS and MS criteria depends on the ultimate tensile strength and the fracture toughness, which are both material properties. Therefore, the critical distance can also be considered a material property independent of the geometry and loading conditions. Equation (35) can be used to calculate the average tangential stress throughout the critical distance:

(35) $$\bar{\sigma_{\theta^{\prime }\theta^{\prime }}}=\frac{1}{d_{c}}\int_{\rho }^{d_{c,\mathrm{VO}}}\sigma_{\theta^{\prime }\theta^{\prime }}\mathrm{dr}$$

Introducing Equation (17) into Equation (35) gives

(36) $$\bar{\sigma_{\theta^{\prime }\theta^{\prime }}}=\frac{1}{\sqrt{2\pi }d_{c}}{\left[{\bar{X}\;K}_{I}^{\mathrm{VO},\rho }\cos^{2}\phi +{\bar{Z}\;K}_{I}^{\mathrm{VO},\rho }\upsilon \sin^{2}\phi -{\bar{Y}\;K}_{\mathrm{III}}^{\mathrm{VO},\rho }\sin2\phi \right]}_{r=\rho }^{r=d_{c,\mathrm{VO}}}$$

where the auxiliary coefficients are

(37) $$\bar{X}=\frac{r^{\lambda_{1}}}{2.38}\left[4.4-{\left(\frac{\rho }{r}\right)}^{2\lambda_{1}}\left(2.8+0.86\left(\frac{\rho }{r}\right)+0.84{\left(\frac{\rho }{r}\right)}^{2}\right)\right]$$

(38) $$\bar{Y}=r^{\lambda_{3}}\left[1.5-1.5{\left(\frac{\rho }{r}\right)}^{2\lambda_{3}}\right]$$

(39) $$\bar{Z}=\frac{r^{\lambda_{1}}}{2.38}\left[7.41-6.22{\left(\frac{\rho }{r}\right)}^{2\lambda_{1}}\right]$$

The process of derivation of the formulas for the VO-MS criterion is similar to that for the VO-MTS criterion. The sole difference is that instead of using the maximum tangential stress at a point, the average tangential stress over a predetermined distance is used. In this regard, the first hypothesis of the VO-MS model can be expressed as

(40) $$\frac{\partial \bar{\sigma_{\theta^{\prime }\theta^{\prime }}}}{\partial \phi }{|}_{\phi =\bar{\phi_{f}}}=0$$

(41) $$\frac{\partial^{2}\bar{\sigma_{\theta^{\prime }\theta^{\prime }}}}{\partial \phi^{2}}{|}_{\phi =\bar{\phi_{f}}}<0$$

where $\bar{\phi_{f}}$ denotes the VO-MS criterion out-of-plane fracture angle. Introducing Equation (36) into Equation (40), and dividing all terms by K_Ic_^VO,ρ^ gives

(42) $$\frac{K_{I}^{\mathrm{VO},\rho }}{K_{\mathrm{Ic}}^{\mathrm{VO},\rho }}\left(\upsilon Z^{\prime \prime }-X^{\prime \prime }\right)\sin2\bar{\phi_{f}}-2\frac{K_{\mathrm{III}}^{\mathrm{VO},\rho }}{K_{\mathrm{Ic}}^{\mathrm{VO},\rho }}Y^{\prime \prime \prime }\cos2\bar{\phi_{f}}=0$$

where

(43) $$X^{\prime \prime }={\left[\frac{r^{\lambda_{1}}}{2.38}\left[4.4-{\left(\frac{\rho }{r}\right)}^{2\lambda_{1}}\left(2.8+0.86\left(\frac{\rho }{r}\right)+0.84{\left(\frac{\rho }{r}\right)}^{2}\right)\right]\right]}_{r=\rho }^{r=d_{c,\mathrm{VO}}}$$

(44) $$Y^{\prime \prime \prime }={\left[r^{\lambda_{3}}\left[1.5-1.5{\left(\frac{\rho }{r}\right)}^{2\lambda_{3}}\right]\right]}_{r=\rho }^{r=d_{c,\mathrm{VO}}}$$

(45) $$Z^{\prime \prime }={\left[\frac{r^{\lambda_{1}}}{2.38}\left[7.41-6.22{\left(\frac{\rho }{r}\right)}^{2\lambda_{1}}\right]\right]}_{r=\rho }^{r=d_{c,\mathrm{VO}}}$$

Like the VO-MTS criterion, and according to Equation (41), the fracture angles of the VO-MS approach under pure mode I loading and pure mode III loading are obtained as 0 and −π⁄4, respectively. Meanwhile, the second assumption of the VO-MS criterion expresses that brittle fracture will occur if the mean tangential stress over d_c,VO_ attains the critical stress of material σ_c_. Mathematically:

(46) $$\frac{1}{\sqrt{2\pi }d_{c}}\left[{X^{\prime \prime }K}_{I}^{\mathrm{VO},\rho }\cos^{2}{\bar{\phi }}_{f}+{Z^{\prime \prime }K}_{I}^{\mathrm{VO},\rho }\upsilon \sin^{2}{\bar{\phi }}_{f}-{Y^{\prime \prime \prime }K}_{\mathrm{III}}^{\mathrm{VO},\rho }\sin2{\bar{\phi }}_{f}\right]=\sigma_{c}$$

Applying the pure mode I fracture conditions (i.e., ${\bar{\phi }}_{f}=0$, K_III_^VO,ρ^ = 0, and K_I_^VO,ρ^ = K_Ic_^VO,ρ^) to Equation (46):

(47) $$\frac{1}{\sqrt{2\pi }d_{c}}K_{\mathrm{Ic}}^{\mathrm{VO},\rho }X^{\prime \prime }=\sigma_{c}$$

Setting Equations (46) and (47) to be identical, and assuming that $Y^{\prime \prime }=Y^{\prime \prime \prime }/d_{c,\mathrm{VO}}^{\lambda_{3}-\lambda_{1}}$:

(48) $$\frac{K_{I}^{\mathrm{VO},\rho }}{K_{\mathrm{Ic}}^{\mathrm{VO},\rho }}\left(X^{\prime \prime }\cos^{2}{\bar{\phi }}_{f}+\upsilon Z^{\prime \prime }\sin^{2}{\bar{\phi }}_{f}\right)-d_{c,\mathrm{VO}}^{\lambda_{3}-\lambda_{1}}\frac{K_{\mathrm{III}}^{\mathrm{VO},\rho }}{K_{\mathrm{Ic}}^{\mathrm{VO},\rho }}Y^{\prime \prime }\sin2{\bar{\phi }}_{f}=X^{\prime \prime }$$

Making an analogy between the VO-MTS and VO-MS criteria, it can be mentioned that analogous fracture limit curves can also be plotted for the VO-MS criterion, considering that the normalized VO-NSIF $r_{c,\mathrm{VO}}^{\lambda_{3}-\lambda_{1}}K_{\mathrm{III}}^{\mathrm{VO},\rho }/K_{\mathrm{Ic}}^{\mathrm{VO},\rho }$ is replaced by $d_{c,\mathrm{VO}}^{\lambda_{3}-\lambda_{1}}K_{\mathrm{III}}^{\mathrm{VO},\rho }/K_{\mathrm{Ic}}^{\mathrm{VO},\rho }$.

### 2.4. Experimental Procedure

**Figure 5:**
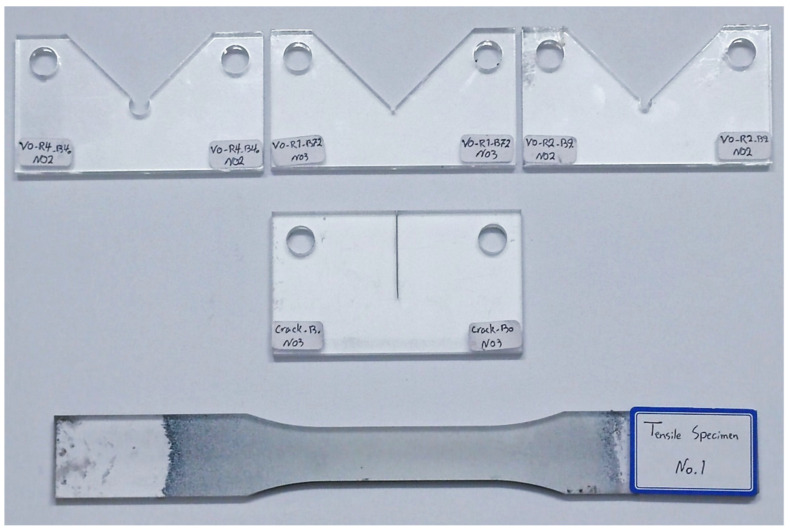
Three types of specimens prepared from 8 mm thick PMMA plates.

In the present study, as illustrated in Figure 5, three types of specimens were prepared, including three tensile test specimens, eight pre-cracked specimens, and forty-five VO-notched specimens. Using a waterjet cutting machine, all samples were obtained from 8 mm thick PMMA plates. Due to the fact that this cold cutting process does not impose significant residual stresses on the specimens, it is an appropriate method for producing the test specimens. Further details about the testing of these specimens are presented below.

#### 2.4.1. Tensile Tests

**Figure 6:**
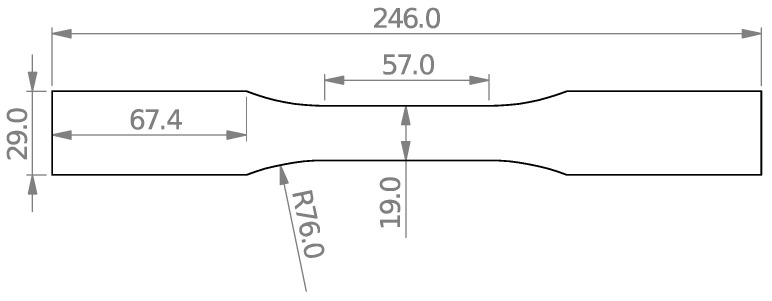
Geometry and dimensions of the tensile test specimens (dimensions in mm).

Although the tensile properties of different PMMA materials are available in the open literature, these properties vary considerably depending on the manufacturer. Therefore, to increase the accuracy of the experimental results, it was more beneficial to obtain the tensile properties of the tested PMMA. For this purpose, as shown in Figure 6, three standard tensile test samples according to ASTM D638 [36] were fabricated. In order to obtain accurate strain values, the digital image correlation (DIC) approach was used. DIC is a non-contact, full-field, and optical technique for measuring displacements/strains on the specimen. This method can determine both the axial and transverse strains. Therefore, Poisson’s ratio can be easily obtained by dividing the transverse strain by the axial strain.

**Figure 7:**
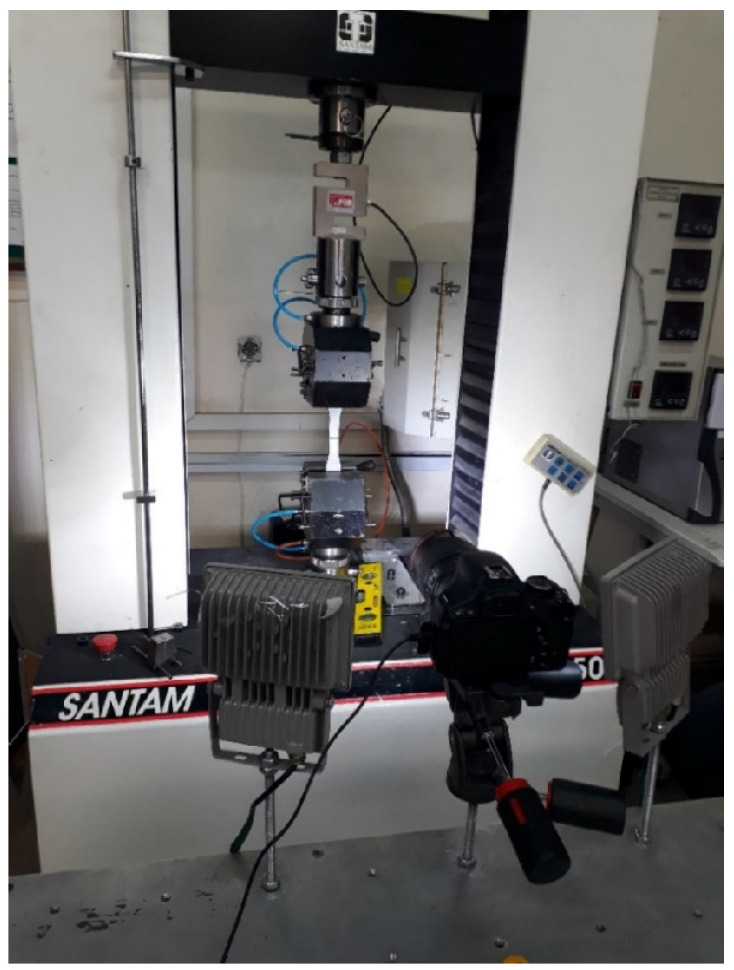
Experimental setup for the tensile tests, including the DIC equipment.

After the waterjet cutting, black and white dots were sprayed over the specimen so that the displacements could be recognized by the DIC software. The laboratory setup included a digital single-lens reflex (DSLR) camera with a micro-lens that allowed for the inspection of the small dots, lighting equipment, a universal tension–compression testing machine, a load cell with a 20 kN capacity, and the specimen, which are all shown in Figure 7.

#### 2.4.2. Fracture Toughness Tests on Cracked Specimens

**Figure 8:**
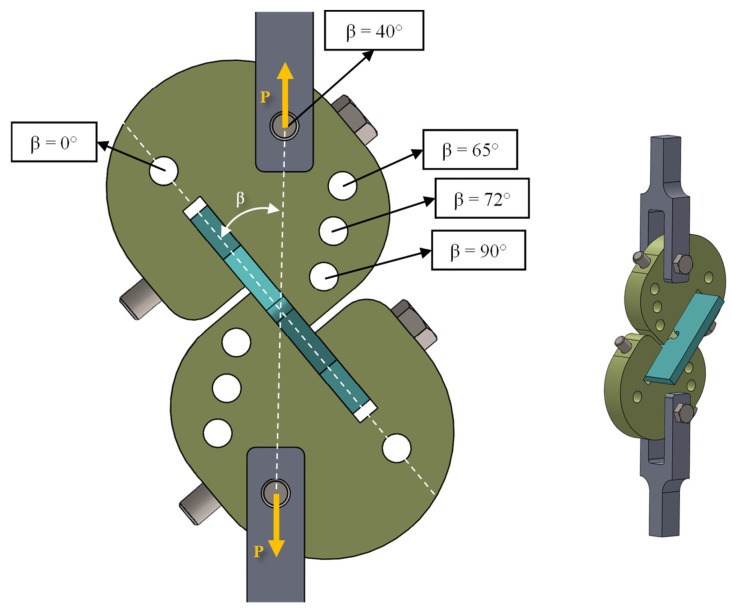
Fixture with ability to create mixed-mode I/III loading conditions from external tensile loads.

In order to create mixed-mode I/III loading conditions on cracked samples by applying only a simple tensile load, the fixture designed by Ayatollahi and Saboori was utilized [28]. As shown in Figure 8, there are five holes in this fixture, each of which can apply mixed-mode I/III loading (with a specific combination of modes I and III) to the specimen. By using the appropriate loading holes, the contributions of the opening and tearing modes can be controlled. The selected loading holes are connected to the fixture by Y-shaped joints, which are placed between the jaws of the tensile test machine. In terms of the loading mode, there was no difference between the geometries of the specimens used in each case, as the different mixed-mode levels were achieved by changing (only) the loading angle β. This issue was one of the advantages of the fixture, which facilitated the process of preparing the specimens. The β angles considered in this experimental campaign and the associated loading modes are given in Table 3. According to the table, to perform the fracture toughness tests on the pre-cracked specimens under pure mode I loading and pure mode III loading, β must be set equal to 0° and 90°, respectively.

**Figure 9:**
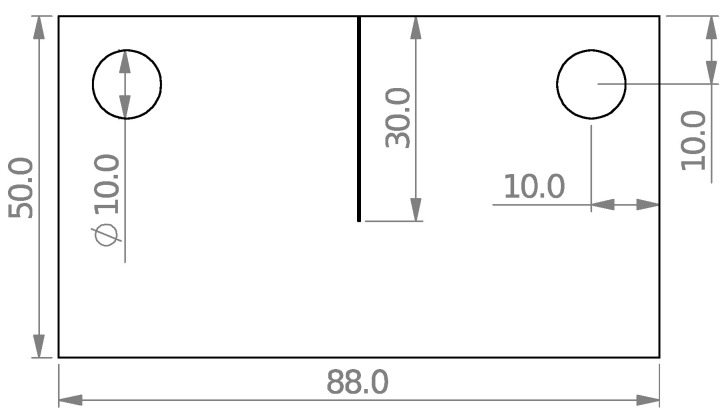
Dimensions (mm) of the 8 mm thick cracked specimens used in fracture toughness tests.

**Figure 10:**
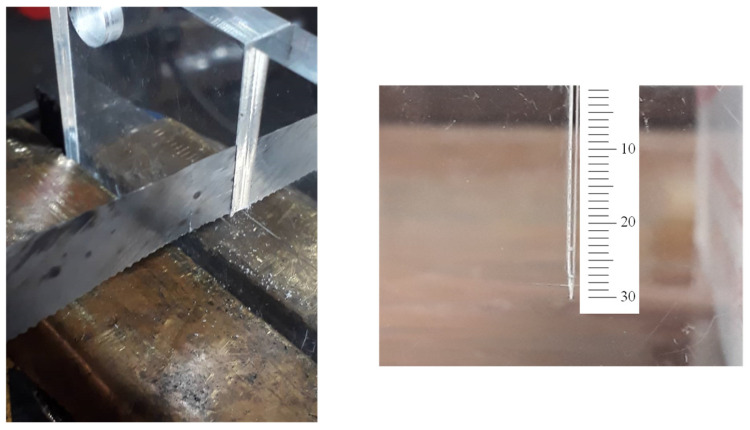
The crack tip sharpening process and the final 30 mm long sharp crack.

The dimensions of the cracked samples are shown in Figure 9. The main challenge in producing the cracked samples was to create a sharp-tip crack, as the notch introduced by the waterjet machine had a round edge with a 0.3 mm tip radius. To address this challenge, a hacksaw with an initial thickness of 0.8 mm was ground to a thickness of 0.1 mm. Following this, the first 28 mm of the crack length was cut with a waterjet process, and the last 2 mm was cut with the ground hacksaw. Figure 10 illustrates this process as well as the finally obtained cracked specimen. In total, eight cracked samples were made, four of which were tested under pure mode I loading, and the rest under pure mode III loading.

#### 2.4.3. Fracture Tests on VO-Notched Specimens

**Figure 11:**
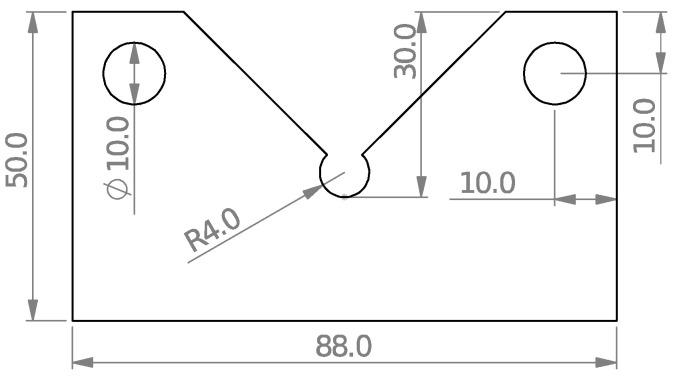
A detailed two-dimensional sketch of a VO-notched sample with 4 mm end-hole radius (all dimensions in mm).

To assess the fracture behavior and to acquire the fracture loads of VO-notched specimens, numerous notched samples are fabricated with various notch end-hole radii, i.e., 1, 2, and 4 mm, and subjected to pure mode I, pure mode III, and mixed-mode I/III loadings. The fixture shown in Figure 8 was also used in these tests. The number of specimens tested was forty-five, considering the three end-hole radii, the five loading angles (β values in Figure 8), and the three repetitions for each test configuration. As an example, the geometry of the VO-notched sample with a 4 mm hole radius is shown in Figure 11.

**Figure 12:**
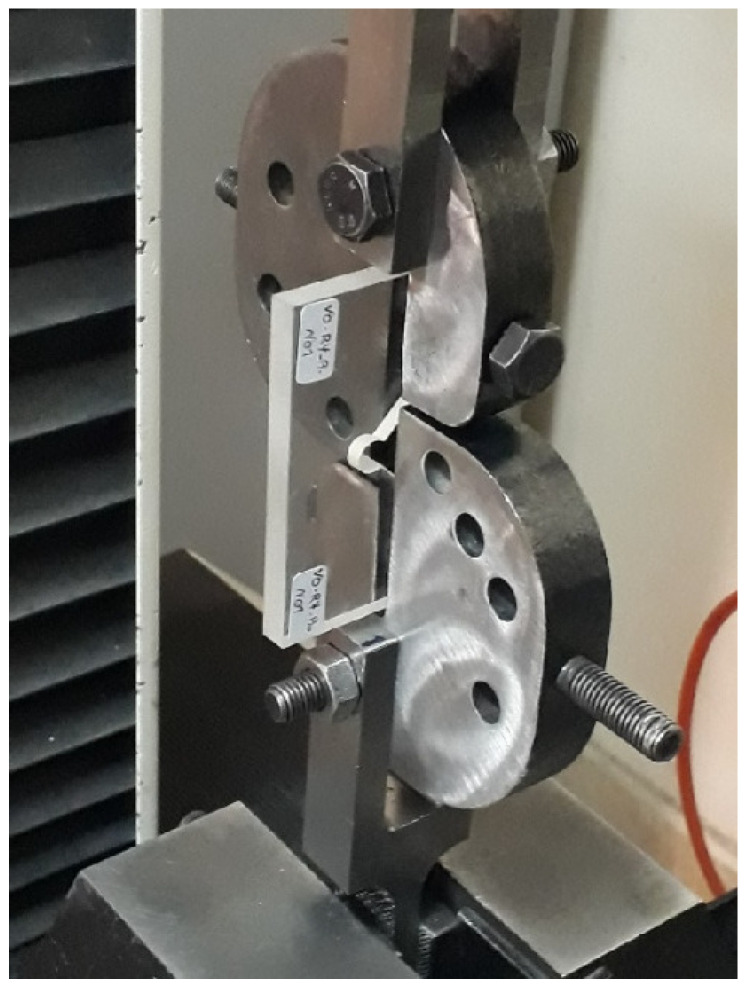
VO-notched specimen with 4 mm end-hole radius under pure mode I loading before testing.

Figure 12 shows the experimental setup for a VO-notched specimen with a 4 mm end-hole radius subjected to pure mode I loading.

### 2.5. Finite Element Analyses

According to Equations (11) and (12), the σ_θθ_ and τ_θz_ values at the notch tip were required in order to calculate the NSIFs at different loading angles. These stresses were numerically obtained by simulating the tested VO-notched polymeric samples in FE software.

The FE models of all test configurations, including the test specimen, fixture, and pins, were generated using the ABAQUS FE software. Throughout this simulation, normal contact between the specimen and the fixture, as well as the pin, was treated as hard contact, and the frictionless tangential contact between the specimen and fixture was assumed to exist due to the smooth surface of the PMMA. In addition, since the fixture components were constructed of high-strength alloy steel and had a much higher stiffness compared to the polymeric samples, they were not considerably deformed during the test and, thus, could be modeled as rigid bodies.

**Figure 13:**
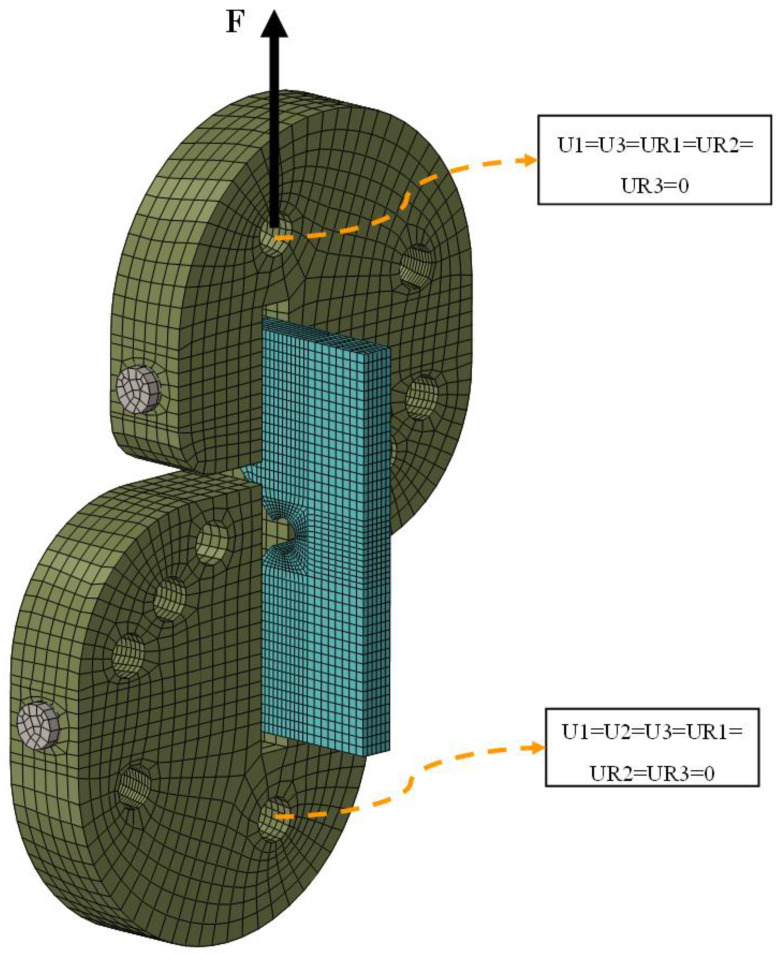
FE model of the test configuration, including the loading and boundary conditions.

Considering the conditions of the fracture experiments conducted in this work, the boundary and loading conditions were also applied to the FE models of the VO-notched samples. A (tensile) force equal to the experiment fracture load was applied to the hole associated with each loading mode (i.e., each β value) in the vertical direction, with the opposite hole being completely fixed, as shown in Figure 13.

**Figure 14:**
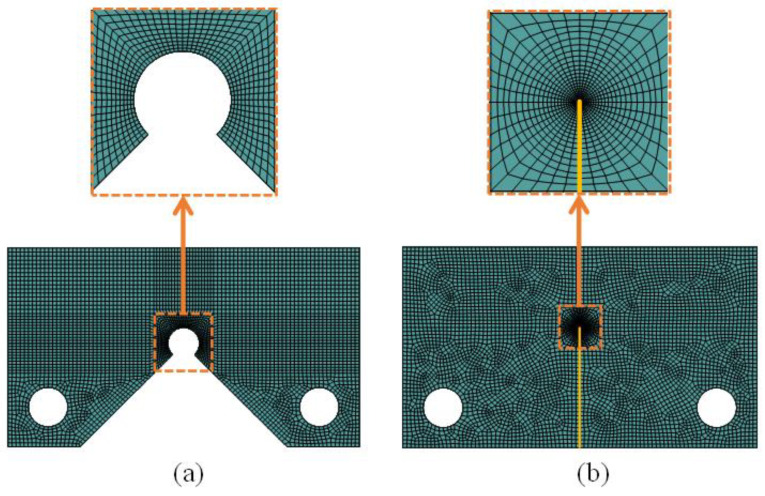
Mesh pattern in (**a**) notched specimen and (**b**) cracked specimen.

Figure 14 shows the mesh patterns of the notched and cracked samples. Due to the presence of cracks and notches, the stress gradient around these stress concentrators was high, and hence, very fine reduced integration quadratic elements were utilized in the vicinity of the stress concentrators, with the aim of significantly increasing the accuracy of the numerical calculations.

## 3. Results

### 3.1. Tensile Tests

**Figure 15:**
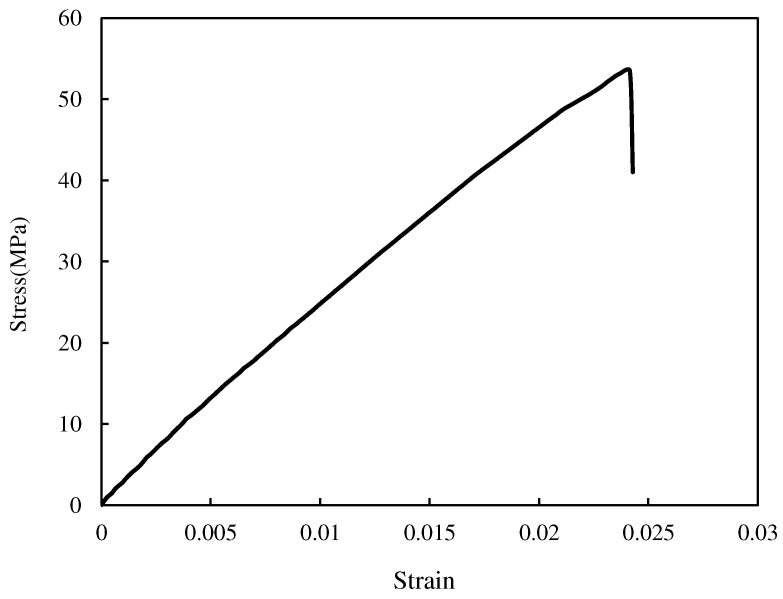
Stress–strain curve of the analyzed PMMA (one of the experimental curves).

**Figure 16:**
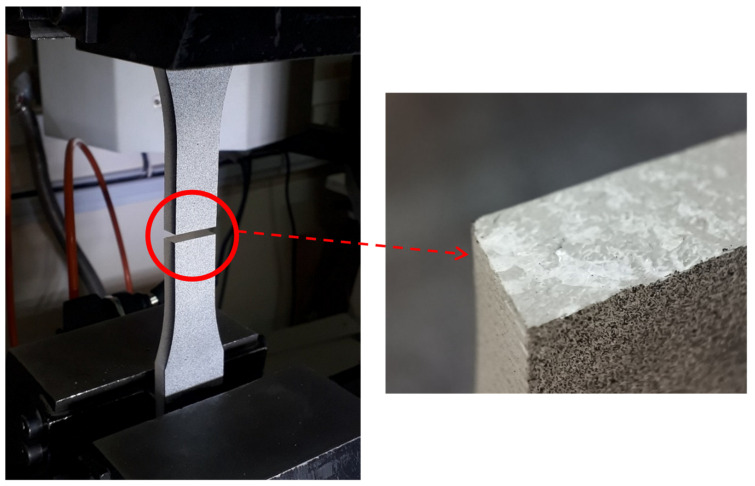
Fracture surface of a broken tensile test sample.

Tensile tests were completed with the absence of any necking during the tests, with sudden fracture (see Figure 15), and the fracture surface perpendicular to the loading direction (see Figure 16), revealing that the tested PMMA was brittle with an almost pure linear elastic behavior. The resulting mechanical properties of the tested PMMA, along with the associated standard deviations, are given in Table 4. It is worth mentioning that the tensile tests were performed on standard tensile samples with three replications.

### 3.2. Fracture Toughness Tests on Cracked Specimens

**Figure 17:**
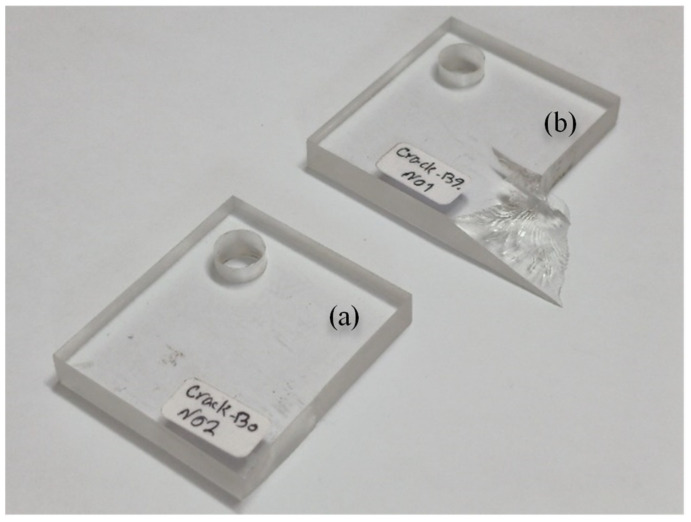
Cracked specimens broken under (**a**) pure mode I loading and (**b**) pure mode III loading.

Figure 17 shows the cracked samples after failure under mode I and mode III loadings, and Table 5 lists the obtained fracture loads. It is evident from the figure that the out-of-plane fracture angle was equal to zero under mode I loading. However, it had a non-zero value under mode III loading.

Finally, the pure mode I and pure mode III fracture toughness (K_Ic_ and K_IIIc_) values of the polymeric material were directly obtained by using the path-independent contour integral method in ABAQUS software. This was carried out by locating the cracked sample shown in Figure 14b inside the fixture shown in Figure 13 and applying the same boundary conditions, but with a force equal to the (average) fracture load achieved in the corresponding fracture toughness in the test (P_avg_ values in Table 5). The resulting mean values of K_Ic_ and K_IIIc_ were 1.62 and 1.78 MPa·m^1/2^, respectively.

### 3.3. Fracture Tests on VO-Notched Specimens

**Figure 18:**
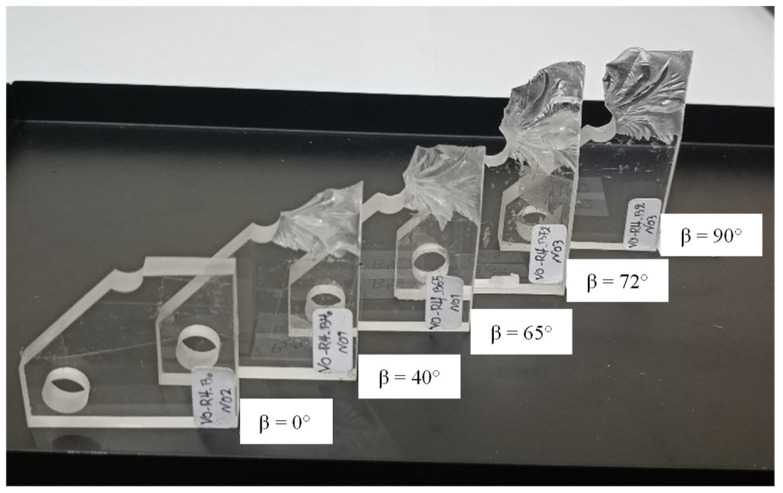
Notched PMMA specimens with 4 mm end-hole radius broken under various combinations of mode I and mode III loadings.

Figure 18 shows the broken specimens corresponding to the 4 mm end-hole radius samples with different loading angles (β). It can be observed that as the tearing mode loading contribution increased, the out-of-plane fracture angle also increased. Table 6 provides the fracture loads of all notched samples. The fracture loads in this table demonstrate that the fracture resistance of notched samples increased as the loading mode changed gradually from pure mode I to pure mode III.

**Figure 19:**
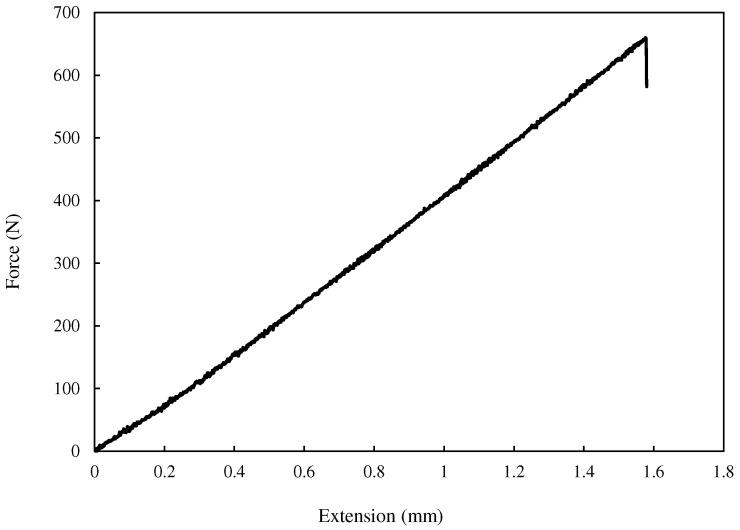
Force–extension curve for a VO-notched polymeric sample with 2 mm end-hole radius subjected to pure mode I loading.

The force–extension curve for a VO-notched sample with a 2 mm end-hole radius subjected to pure mode I loading is shown in Figure 19. The linear behavior observed in this case was also observed in the rest of the specimens. Thus, LENFM models, such as the VO-MTS and VO-MS criteria described above in Section 2, can be employed here to estimate the mixed-mode I/III notch fracture toughness (NFT) of the VO-notched polymeric specimens tested in this research.

### 3.4. NSIF Predictions and Comparison with Experimental Results

In order to verify the agreement between the theoretical predictions of the two fracture criteria outlined in Section 2 and the experimental results provided above, the VO-NSIFs associated with the test data were computed and compared with the fracture limit curves of the VO-MTS and VO-MS criteria.

The calculation of the VO-NSIFs related to each fracture test required applying the corresponding fracture load reported in Table 6 to the FE model described in Section 2.5. Then, by extracting the tangential and out-of-plane shear stresses at the notch tip from the linear elastic FE analysis, the critical VO-NSIFs were computed using Equations (11) and (12). As explained in Section 2, the fracture limit curves were plotted in a plane with nondimensional X and Y axes. To compare the critical VO-NSIFs achieved from the experimental results with the fracture limit curves, they had to be divided by the mode I notch fracture toughness value (K_Ic_^VO,ρ^), which depended on the notch end-hole radius. Table 7 lists the values of K_Ic_^VO,ρ^ for various notch end-hole radii as well as those of the critical distances for the VO-MTS and VO-MS criteria.

**Figure 20:**
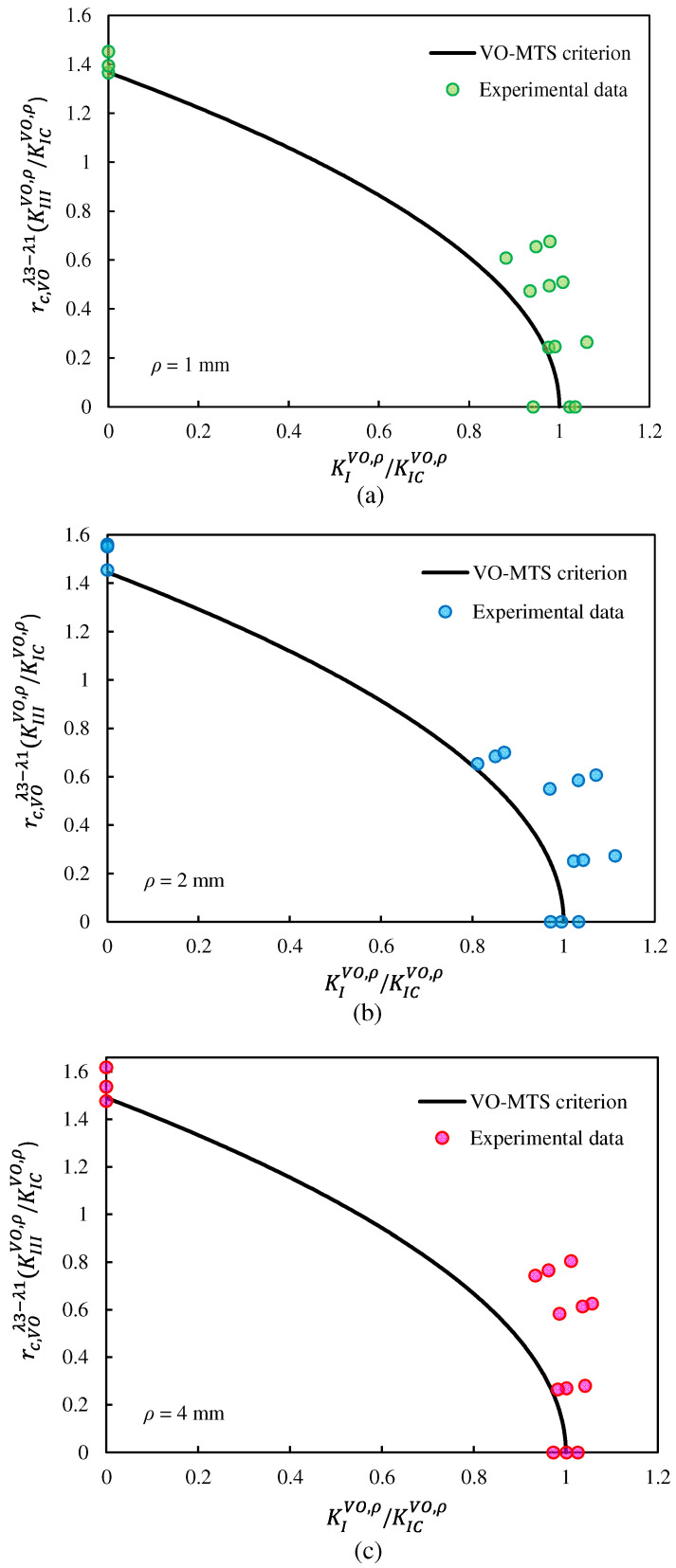
Fracture limit curves derived from the VO-MTS criterion, and comparison with the experimental data for notch end-hole radii of: (**a**) 1 mm, (**b**) 2 mm, (**c**) and 4 mm.

**Figure 21:**
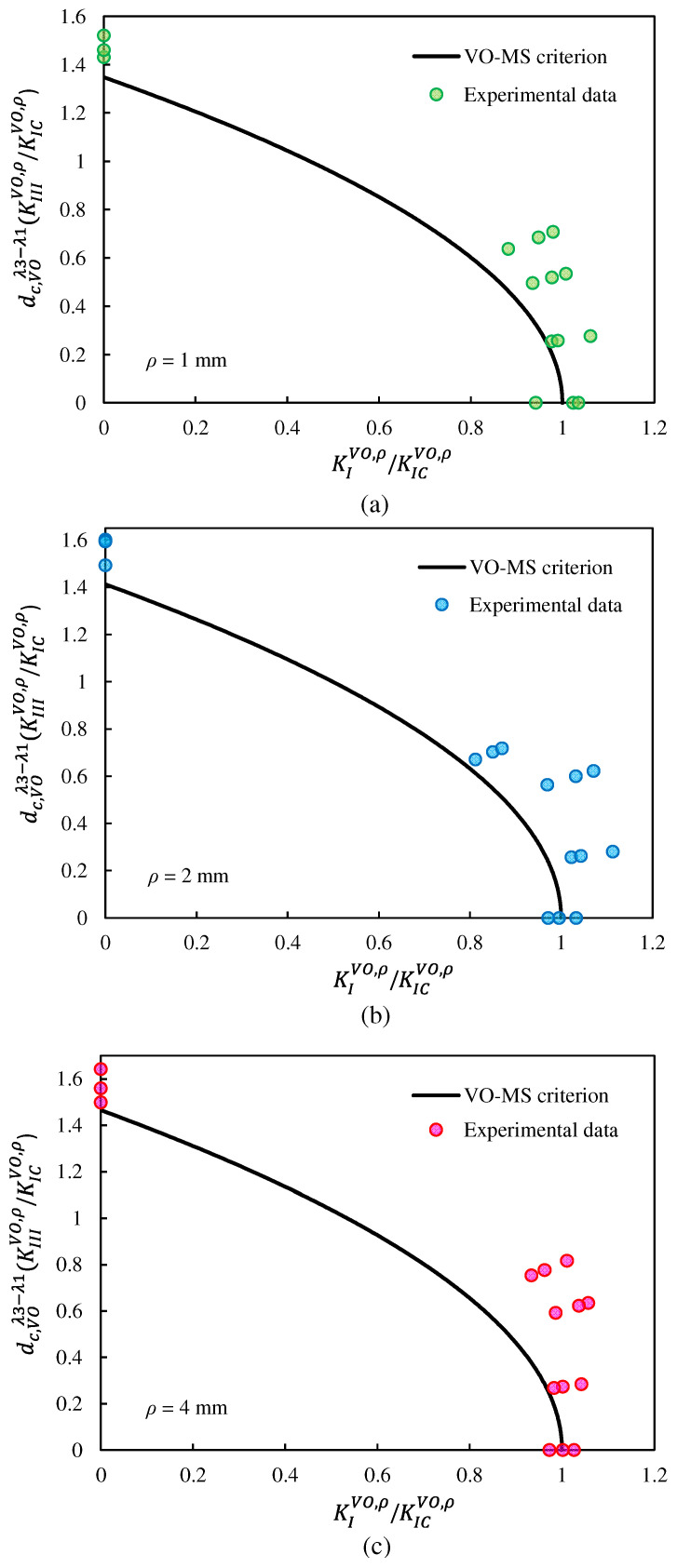
Fracture limit curves derived from the VO-MS criterion, and comparison with the experimental data for notch end hole radii of: (**a**) 1 mm, (**b**) 2 mm, (**c**) and 4 mm.

Figure 20 and Figure 21 show the fracture limit curves of the VO-MTS and VO-MS criteria, respectively, including the experimental data associated with the VO-notched polymeric samples tested. In this research, in addition to pure mode I and pure mode III, three combined modes were also tested. However, in order to cover more points of the fracture limit curves, it was necessary to perform more tests for other loading angles.

## 4. Discussion

It can be qualitatively seen in Figure 20 and Figure 21 that both fracture criteria successfully predicted the experimental data. In spite of this, a parameter called the normalized effective NSIF (NENSIF) was proposed and employed to compare the theoretical predictions with the experimental data quantitatively. Equations (49) and (50) provide the nondimensional expressions of the NENSIF for the VO-MTS and VO-MS criteria, respectively.

(49) $$K_{eff,MTS}^{VO,\rho }=\sqrt{{\left(\frac{K_{I}^{VO,\rho }}{K_{Ic}^{VO,\rho }}\right)}^{2}+{\left(r_{c,VO}^{\lambda 3-\lambda 1}\frac{K_{III}^{VO,\rho }}{K_{Ic}^{VO,\rho }}\right)}^{2}}$$

(50) $$K_{eff,MS}^{VO,\rho }=\sqrt{{\left(\frac{K_{I}^{VO,\rho }}{K_{Ic}^{VO,\rho }}\right)}^{2}+{\left(d_{c,VO}^{\lambda 3-\lambda 1}\frac{K_{III}^{VO,\rho }}{K_{Ic}^{VO,\rho }}\right)}^{2}}$$

Table 8 reports the values of the theoretical NENSIF derived from the VO-MTS criterion and compares them with those corresponding to the experimental results, with the corresponding discrepancies included. Similar data are also reported in Table 9 for the VO-MS criterion.

As shown in Table 8 and Table 9, the overall mean discrepancies between the predictions of the VO-MTS and VO-MS criteria and the experimental data are equal to 8.0% and 9.7%, respectively, which indicates the success of both theoretical criteria in predicting the fracture of VO-notched polymeric samples exposed to combined tension–torsion loading. It is possible to attribute a considerable portion of the whole difference between the experimental data and theoretical estimations to the errors that occurred due to an imperfect preparation of the test samples as well as those happening during the experiments. For example, as shown in Figure 8, the test configuration was composed of several components. Ideally, it could be assumed that all these components were perfectly connected to each other and that there was no slip between them. Thus, it could be assumed that the load was completely transferred from the testing machine to the notched specimen. However, due to clearances that existed between the components, the load externally applied to the test configuration may not have been fully transferred to the notched sample, leading to a possible increase in the experimentally acquired fracture load.

As another source of discrepancy between the theoretical and experimental results, the coupling of the mode III and II loadings, which was neglected in the present study, can be mentioned. According to [37,38,39], pure mode II and pure mode III loadings cannot exist independently because of the presence of Poisson’s ratio and also the mutual effects of the in-plane and out-of-plane deformations of the cracked and notched components. Although this study did not involve mode II loading directly, mode III loading induced some amount of in-plane shear deformations to the VO-notch, leading to the creation of mode II loading.

**Figure 22:**
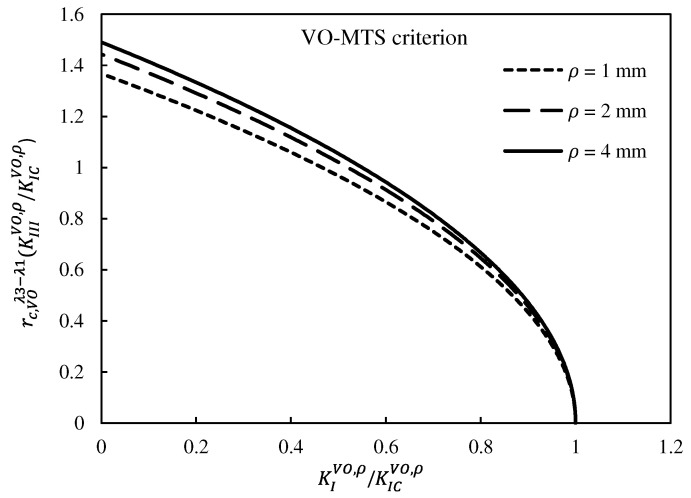
VO-MTS criterion fracture limit curves for different notch end-hole radii.

**Figure 23:**
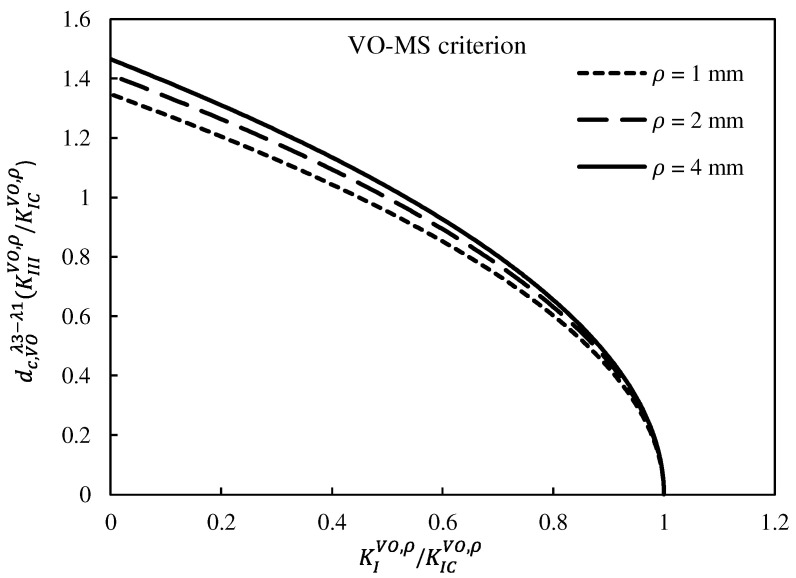
VO-MS criterion fracture limit curves for different notch end-hole radii.

To investigate the influence of the notch end-hole radius on the predictions of the theoretical models, the fracture limit curves of VO-MTS and VO-MS criteria for different radii are plotted in Figure 22 and Figure 23, respectively. These figures illustrate that as the size of the hole at the end of the notch increased, the area of the safe zone of the plot increased for both theoretical models. This is because of the corresponding decrease in the stress concentration, leading to an increase in the fracture load. This issue can also be seen in Table 6 regarding the experimentally achieved fracture loads of the VO-notched polymeric specimens.

**Figure 24:**
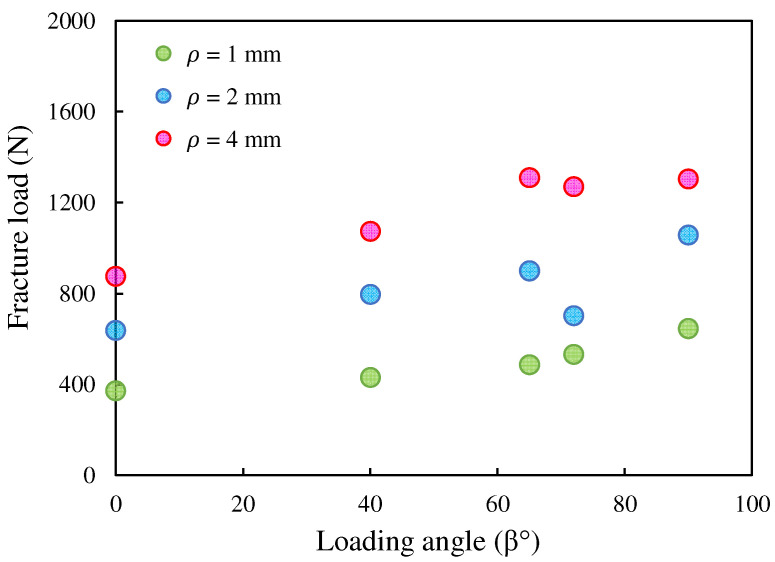
Experimental failure load values at different loading angles and different notch end-hole radii.

Figure 24 shows the effect of the notch radius on the experimental fracture load. As it is clear from this figure, at all loading angles, the failure load increased with the notch radius. In addition, it can be seen that by increasing the loading angle, i.e., by enhancing the participation of the tearing mode, the fracture resistance of the samples has risen.

## 5. Conclusions

By means of the linear elastic stress distributions around VO-notches under various loading conditions, a formulation was derived for the distribution of tangential stress under mixed-mode I/III loading. This formulation was used to establish the VO-MTS and VO-MS criteria and to obtain the associated NSIF fracture limit curves for the different notch end-hole radii analyzed here.

It was found that the limit curves of both criteria were very close to each other, implying that both criteria provided almost identical notch fracture toughness values under mixed-mode I/III loading. To evaluate the validity of the curves, a new and extensive experimental campaign was conducted, in which numerous VO-notched samples with different end-hole radii were made from PMMA and subjected to pure opening mode loading, combined opening–tearing mode loading, and pure tearing mode loading by means of a special fixture.

A comparison between the experimental and the theoretical results showed that both the VO-MTS and VO-MS models accurately predicted the failure of brittle VO-notched specimens exposed to combined tension–torsion loading conditions. Since there were no significant differences between the results of the two criteria, using the VO-MTS criterion is preferable due to the simplicity of its mathematical formulation.

The presence of the fixture with different parts, having clearances between each other and causing indirect and imperfect load transfer from the testing machine to the notched specimen, is recognized as a possible reason for the discrepancy between the experimental and theoretical results, together with the interaction between the loading modes.

## Figures and Tables

**Table 1:** Values of the constant and auxiliary parameters of the stress field equations for VO-notches.

Parameter	Value
$\lambda_{1}$	0.5445
$\lambda_{3}$	$\frac{\pi }{2\left(\pi -\alpha \right)}=\frac{2}{3}$
$\varphi_{1}\left(\gamma \right)$	0.8388
$\psi_{12}\left(0\right)\chi_{11}\left(0\right)$	1.33
$\psi_{11}\left(0\right)$	1.13

**Table 2:** Values of notch stress intensity factors and fracture angle under pure mode I and pure mode III loading conditions.

Loading Mode	$K_{I}^{VO,\rho }$	$K_{III}^{VO,\rho }$	$\phi_{f}$
Pure mode I	$K_{\mathrm{IC}}^{\mathrm{VO},\rho }$	0	0
Pure mode III	0	$K_{\mathrm{IIIC}}^{\mathrm{VO},\rho }$	±π/4

**Table 3:** Values of β for the different loading modes.

β (°)	Loading Mode
0	Pure mode I
40, 65, 72	Mixed mode I/III
90	Pure mode III

**Table 4:** Tensile properties of the PMMA analyzed in this work.

Material Property	Value	Standard Deviation
Elastic modulus (GPa)	2.45	0.05
Ultimate tensile strength (MPa)	53.5	0.61
Poisson’s ratio	0.4	0.003

**Table 5:** Pure mode I and pure mode III fracture loads of the cracked PMMA samples.

Loading Mode	Fracture Load (N)				
	$P_{1}$	$P_{2}$	$P_{3}$	$P_{4}$	$P_{avg}$
Pure mode I	297.4	256.5	278.1	276.3	277.1
Pure mode III	431.2	482.8	453.5	488.7	464.1

**Table 6:** Fracture loads of the VO-notched PMMA specimens at different loading angles.

Notch End-Hole Radius (mm)	Loading Angle	Fracture Load (N)			
	β (°)	$P_{1}$	$P_{2}$	$P_{3}$	$P_{avg}$
1	0 (mode I)	379.8	384.2	349.5	371.2
	40	415.3	451.4	421.1	429.3
	65	467.2	503.5	488.3	486.3
	72	556.6	501.3	538.7	532.2
	90 (mode III)	628.1	641.0	667.5	645.5
2	0 (mode I)	658.1	634.4	619.1	637.2
	40	767.5	783.1	835.6	795.4
	65	851.7	906.6	940.6	899.6
	72	706.7	674.9	723.1	701.6
	90 (mode III)	1009.8	1083.1	1077.3	1056.7
4	0 (mode I)	897.6	851.0	875.9	874.8
	40	1066.4	1046.2	1109.7	1074.1
	65	1347.8	1321.7	1257.3	1308.9
	72	1222.8	1324.2	1260.0	1269.0
	90 (mode III)	1297.9	1366.3	1247.1	1303.8

**Table 7:** Values of K_Ic_^VO,ρ^ for different notch end-hole radii, as well as those of the critical distances for VO-MTS and VO-MS criteria.

ρ (mm)	K_Ic_^VO,ρ^ (MPa·m^0.45^)	r_c,VO_ (mm)	d_c,VO_ (mm)
1	2.6	1.17	1.7
2	4.46	2.17	2.7
4	6.37	4.17	4.7

**Table 8:** Comparison of the NENSIF values obtained from the fracture tests on notched polymeric samples with those predicted by the VO-MTS criterion.

ρ (mm)	Loading Angle, β (°)	Mean Experimental NENSIF	Predicted NENSIF	Discrepancy (%)
1	0 (mode I)	1	1	0
	40	1.04	1.0	3.8
	65	1.09	1.0	8.4
	72	1.14	1.0	11.6
	90 (mode III)	1.40	1.37	2.7
				**Avg.: 6.6**
2	0 (mode I)	1	1	0
	40	1.09	1.0	8.0
	65	1.18	1.01	13.9
	72	1.08	1.03	4.7
	90 (mode III)	1.52	1.44	5.1
				**Avg.: 7.9**
4	0 (mode I)	1	1	0
	40	1.04	1.0	3.8
	65	1.19	1.02	14.2
	72	1.24	1.04	15.7
	90 (mode III)	1.54	1.49	4.0
				**Avg.: 9.4**
				**Avg.: 8.0**

**Table 9:** Comparison of the NENSIF values obtained from the fracture tests on notched polymeric samples with those predicted by the VO-MS criterion.

ρ (mm)	Loading Angle, β (°)	Mean Experimental NENSIF	Predicted NENSIF	Discrepancy (%)
1	0 (mode I)	1	1	0
	40	1.04	1.0	4.2
	65	1.1	1.0	9.5
	72	1.16	1.0	13.2
	90 (mode III)	1.47	1.35	8.4
				**Avg.: 8.8**
2	0 (mode I)	1	1	0
	40	1.09	1.0	8.3
	65	1.19	1.01	14.8
	72	1.10	1.02	6.5
	90 (mode III)	1.56	1.41	9.6
				**Avg.: 9.8**
4	0 (mode I)	1	1	0
	40	1.04	1.0	4.0
	65	1.20	1.02	14.9
	72	1.25	1.04	16.8
	90 (mode III)	1.57	1.46	6.4
				**Avg.: 10.5**
				**Avg.: 9.7**

## Data Availability

The data presented in this study are available upon request from the corresponding authors.
